# A Subgroup Analysis of Perioperative Pembrolizumab in Clinical Stage II Non-Small-Cell Lung Cancer from the Randomized KEYNOTE-671 Study

**DOI:** 10.1093/ejcts/ezag028

**Published:** 2026-03-24

**Authors:** Masahiro Tsuboi, Heather Wakelee, Marina C Garassino, Shugeng Gao, Alexander Luft, Ke-Neng Chen, Jonathan D Spicer, Yuming Zhu, Hisashi Saji, Morihito Okada, Tõnu Vanakesa, Haiquan Chen, Guofang Zhao, Norihiko Ikeda, David R Jones, Benny Weksler, Chien-Sheng Huang, Erin Jensen, Steven M Keller, Ayman Samkari, Moishe Liberman

**Affiliations:** Department of Thoracic Surgery, National Cancer Center Hospital East, Kashiwa, Chiba 277-8577, Japan; Department of Medicine, Stanford University School of Medicine/Stanford Cancer Institute, Stanford, CA 94305, United States; Department of Medicine, University of Chicago Medicine and Biological Sciences, Chicago, IL 60637, United States; Department of Thoracic Surgery, National Cancer Center/National Clinical Research Center for Cancer/Cancer Hospital, Chinese Academy of Medical Sciences and Peking Union Medical College, Beijing 100021, China; Department of Oncology No. 1 (Thoracic Surgery), SBHI Leningrad Regional Clinical Hospital, St Petersburg 194291, Russia; State Key Laboratory of Molecular Oncology, The First Department of Thoracic Surgery, Peking University Cancer Hospital & Institute, Beijing 100142, China; Department of Surgery, McGill University Health Centre, Montreal, QC H4G 1A4, Canada; Thoracic Surgery Department, Shanghai Pulmonary Hospital, Shanghai 200433, China; Department of Chest Surgery, St Marianna University School of Medicine Hospital, Kawasaki 216-8511, Japan; Department of Surgical Oncology, Hiroshima University Hospital, Hiroshima 734-8551, Japan; Department of Thoracic Surgery, North Estonia Medical Centre, Tallinn 13419, Estonia; Department of Thoracic Surgery and State Key Laboratory of Genetics and Development of Complex Phenotypes, Fudan University Shanghai Cancer Center, Shanghai 200032, China; Department of Cardiothoracic Surgery, Hwa Mei Hospital, University of Chinese Academy of Sciences (Ningbo No. 2 Hospital), Ningbo 315010, China; Department of Surgery, Tokyo Medical University Hospital, Tokyo 160-0023, Japan; Department of Surgery, Memorial Sloan Kettering Cancer Center, Weill Cornell Medical College, New York, NY 10065, United States; Department of Thoracic and Cardiovascular Surgery, Allegheny General Hospital, Pittsburgh, PA 15212, United States; Division of Thoracic Surgery, Department of Surgery, Taipei Veterans General Hospital, Taipei 112201, Taiwan; School of Medicine, National Yang-Ming Chiao-Tung University, Taipei 30010, Taiwan; Biostatistics and Research Decision Sciences, Merck & Co., Inc., Rahway, NJ 07065, United States; Global Clinical Development, Merck & Co., Inc., Rahway, NJ 07065, United States; Global Clinical Development, Merck & Co., Inc., Rahway, NJ 07065, United States; Division of Thoracic Surgery, Centre Hospitalier de l’Université de Montréal (CHUM), University of Montreal, Montreal, QC H3A 2B4, Canada

**Keywords:** perioperative therapy, non-small-cell lung cancer, pembrolizumab

## Abstract

**Objectives:**

Perioperative pembrolizumab plus neoadjuvant chemotherapy significantly improved outcomes versus neoadjuvant chemotherapy alone in early-stage, resectable, non-small-cell lung cancer (NSCLC) in the phase 3 KEYNOTE-671 study. We report outcomes in participants with baseline clinical stage II disease.

**Methods:**

Participants with untreated, resectable, stage II-IIIB (N2) NSCLC were randomized 1:1 to receive pembrolizumab 200 mg or placebo every 3 weeks plus chemotherapy for 4 cycles, followed by surgery and adjuvant pembrolizumab or placebo for 13 cycles (∼9 months). Exploratory analyses were performed in participants with clinical stage II disease.

**Results:**

Of 797 randomized participants, 239 had stage II disease (pembrolizumab plus chemotherapy, *n* = 118; chemotherapy only, *n* = 121). Median study follow-up at data cutoff (August 19, 2024) was 49.9 (range, 32.2-75.3) months. Among participants who underwent surgery, 94/99 (94.9%) had R0 resections in the pembrolizumab arm and 89/103 (86.4%) in the neoadjuvant chemotherapy only arm. Event-free survival (hazard ratio [HR], 0.50; 95% CI, 0.34-0.74), overall survival (HR, 0.69; 95% CI, 0.43-1.11), major pathological response (difference, 25.7%; 95% CI, 15.3-35.9), and pathological complete response (difference, 21.3%; 95% CI, 13.2-30.2) were improved in the pembrolizumab arm. Grade 3-4 treatment-related adverse events (AEs) occurred in 50.0% of participants treated with pembrolizumab plus chemotherapy and 40.5% with chemotherapy; no treatment-related AEs led to death.

**Conclusions:**

In participants with stage II NSCLC, perioperative pembrolizumab improved efficacy outcomes with manageable safety versus neoadjuvant chemotherapy alone, consistent with the overall KEYNOTE-671 population. These results support the use of this regimen in patients with stage II disease.

**Trial Registration:**

ClinicalTrials.gov, NCT03425643, registered/first posted on February 7, 2018 (https://www.clinicaltrials.gov/study/NCT03425643).

## Graphical Abstract

**Figure:**
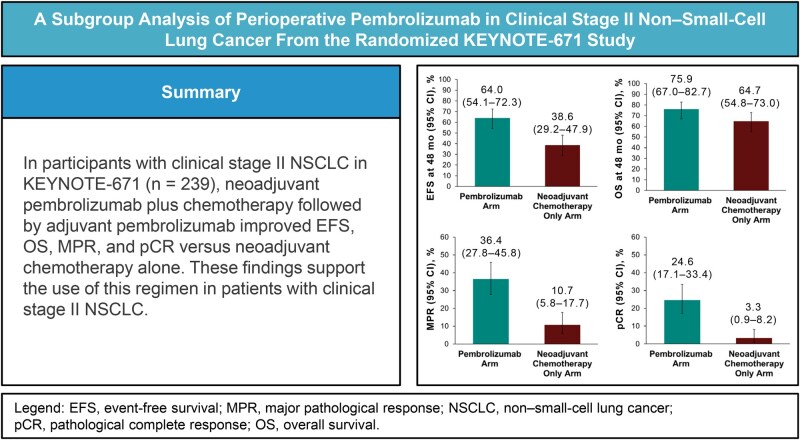

## Introduction

The addition of perioperative anti-programmed cell death protein 1 or anti-programmed cell death ligand 1 (anti-PD-[L]1) therapy to neoadjuvant chemotherapy has improved outcomes versus neoadjuvant chemotherapy alone in patients with early-stage, resectable, non-small-cell lung cancer (NSCLC).^1–5^ However, questions remain as to whether patients with clinical stage II disease receive the same benefit from these treatments as those with stage III disease. Studies evaluating perioperative^3^ or neoadjuvant^6^ immunotherapy plus chemotherapy have shown a smaller magnitude of event-free survival (EFS) benefit in participants with stage IB to II (per American Joint Committee on Cancer [AJCC], seventh edition) or stage II (AJCC, eighth edition) versus stage III NSCLC. There is also concern that these patients may forego surgery after neoadjuvant therapy.^7^

In the phase 3 KEYNOTE-671 study, neoadjuvant pembrolizumab (anti-PD-1) plus chemotherapy followed by surgery and adjuvant pembrolizumab significantly improved outcomes versus neoadjuvant chemotherapy and surgery alone in participants with resectable stage II, IIIA, or IIIB (N2) NSCLC,^1^^,^^8^ including EFS (hazard ratio [HR], 0.58; 95% CI, 0.46-0.72; *P *< .001), major pathological response (MPR; percentage difference, 19.2; 95% CI, 13.9-24.7; *P *< .0001) and pathological complete response (pCR; percentage difference, 14.2; 95% CI, 10.1-18.7; *P *< .0001) at interim analysis (IA) 1, and overall survival (OS; HR, 0.72; 95% CI, 0.56-0.93; *P *= .0052) at IA2.^8^ EFS and OS benefits of pembrolizumab were maintained at IA3.^9^ The safety profile of pembrolizumab in KEYNOTE-671 was manageable and consistent with the known profile of the combination therapy.^8^

We present an exploratory analysis at IA3 (median follow-up, 49.9 months) in a prespecified subgroup of participants in KEYNOTE-671 with clinical stage II disease at baseline.

## Methods

### Study design and participants

The KEYNOTE-671 (NCT03425643) study design has been described previously.^1^ Briefly, KEYNOTE-671 was a randomized, double-blind, phase 3 study of participants aged ≥18 years with previously untreated, pathologically confirmed resectable stage II, IIIA, or IIIB (N2) NSCLC (AJCC eighth edition). Participants provided a tumour tissue sample for PD-L1 testing, had an Eastern Cooperative Oncology Group performance status of 0 or 1, and had adequate organ function.

The study was approved by the appropriate institutional review boards and regulatory agencies and was conducted in accordance with ethical principles that have their origin in the Declaration of Helsinki and are consistent with the principles of Good Clinical Practice.^8^ All participants provided written informed consent before entering the trial.

### Treatment

Participants were randomized 1:1 to receive ≤4 cycles of neoadjuvant pembrolizumab 200 mg or placebo every 3 weeks (Q3W) with chemotherapy (cisplatin 75 mg/m^2^ on day 1 of every 3-week cycle plus either gemcitabine 1000 mg/m^2^ on days 1 and 8 [squamous tumours] or pemetrexed 500 mg/m^2^ on day 1 [nonsquamous tumours]). Following surgery, participants received up to 13 cycles (∼9 months) of adjuvant pembrolizumab 200 mg (pembrolizumab arm) or placebo (neoadjuvant chemotherapy only arm) Q3W. Treatment continued until the maximum number of doses was reached, disease progression or recurrence, unacceptable toxicity, participant withdrawal, or when any other criterion for treatment discontinuation was met. Randomization was stratified by clinical stage (II vs III), PD-L1 tumour proportion score (TPS; <50% vs ≥50%), histology (squamous vs nonsquamous), and geographic region (East Asia vs not East Asia).

Surgery was performed ≤20 weeks after the first neoadjuvant dose; participants who received <4 cycles of neoadjuvant therapy underwent surgery within 4-8 weeks of the last neoadjuvant dose. Additional details of study treatment are available in **Supplementary Materials**.

### Endpoints

Dual primary endpoints were EFS (time from randomization to first radiographic disease progression per Response Evaluation Criteria in Solid Tumors version 1.1, local progression precluding planned surgery, inability to resect the tumour, investigator-assessed local or distant recurrence, or death from any cause) and OS (time from randomization to death from any cause). Secondary endpoints included MPR (≤10% viable tumour cells in the resected primary tumour and lymph nodes) and pCR (absence of residual invasive cancer in the resected lung specimen and lymph nodes, ie, ypT0/Tis ypN0) as assessed by blinded central pathologist review, and safety.

### Assessments

Tumour imaging was performed by computed tomography or magnetic resonance imaging at screening during the neoadjuvant phase per the number of neoadjuvant treatment cycles received: 3 weeks after completion of cycles 2 and 4 (participants who received 4 cycles), 3 weeks after cycle 2 and 4 weeks after cycle 3 (3 cycles), 3 weeks after cycle 2 (2 cycles), or 3 weeks after cycle 1 (1 cycle). After surgery, imaging was performed ≤4 weeks before starting adjuvant therapy and every 16 weeks during the adjuvant phase.

Adverse events (AEs) were assessed throughout the study and for 30 days after discontinuation of study treatment (90 days for serious AEs). Adverse events were graded per National Cancer Institute Common Terminology Criteria for Adverse Events version 4.03. Immune-mediated AEs and infusion reactions were based on a list of preferred terms intended to capture known risks of pembrolizumab and were considered regardless of attribution to study treatment by the investigator.

PD-L1 expression was centrally assessed in tumour tissue samples using PD-L1 IHC 22C3 pharmDx (Agilent Technologies, Carpinteria, CA, USA).

### Statistical analyses

This was an exploratory analysis of a protocol-specified subgroup of participants with clinical stage II disease at baseline. Efficacy was assessed in all randomized participants and safety in all randomized participants who received ≥1 dose of study treatment.

Hazard ratios and 95% CIs for EFS and OS were estimated from an unstratified Cox proportional hazard model with Efron’s method of tie handling and treatment as a covariate. Event-free survival and OS estimates were based on the Kaplan-Meier method. For MPR and pCR, treatment differences and 95% CIs were calculated using the unstratified Miettinen and Nurminen method. The analyses were not controlled for multiplicity and are descriptive only. Participants with missing data for EFS or OS were censored at the last disease assessment or last known date the participant was alive, respectively. Participants with missing data for MPR or pCR were considered nonresponders.

## Results

### Participants

Overall, 797 participants were randomized; 239 participants (30.0%) had stage II disease at baseline and were included in this analysis (pembrolizumab arm, *n* = 118; neoadjuvant chemotherapy only arm, *n* = 121). In the respective arms, 22 (18.6%) and 19 participants (15.7%) had stage IIA disease, and 96 (81.4%) and 102 (84.3%) had stage IIB disease. Baseline participant demographics and disease characteristics were similar between treatment arms (**Table 1**). In the pembrolizumab and neoadjuvant chemotherapy only arms, 74.6% and 73.6% of participants were male, respectively, with a median (range) age of 63 (26-79) and 63 (45-79) years.

**Table 1.** Participant Baseline Characteristics

Characteristics	**Pembrolizumab arm** **(*n* = 118)**	**Neoadjuvant chemotherapy only arm** **(*n* = 121)**
Age, median (range), y	63 (26-79)	63 (45-79)
Sex		
Female	30 (25.4)	32 (26.4)
Male	88 (74.6)	89 (73.6)
Race		
Asian	52 (44.1)	54 (44.6)
Black or African American	1 (0.8)	3 (2.5)
Multiple	2 (1.7)	3 (2.5)
White	58 (49.2)	57 (47.1)
Missing data	5 (4.2)	4 (3.3)
Geographic region		
East Asia	51 (43.2)	52 (43.0)
Not East Asia	67 (56.8)	69 (57.0)
ECOG PS		
0	80 (67.8)	76 (62.8)
1	38 (32.2)	45 (37.2)
Histology		
Squamous	66 (55.9)	64 (52.9)
Nonsquamous	52 (44.1)	57 (47.1)
Smoking status		
Current	30 (25.4)	34 (28.1)
Former	71 (60.2)	74 (61.2)
Never	17 (14.4)	13 (10.7)
Clinical stage		
IIA	22 (18.6)	19 (15.7)
IIB	96 (81.4)	102 (84.3)
Clinical tumour stage		
T1	9 (7.6)	11 (9.1)
T2	46 (39.0)	44 (36.4)
T3	63 (53.4)	66 (54.5)
Clinical node status		
N0	85 (72.0)	85 (70.2)
N1	33 (28.0)	36 (29.8)
PD-L1 TPS		
≥50%	36 (30.5)	34 (28.1)
1%-49%	39 (33.1)	36 (29.8)
<1%	43 (36.4)	51 (42.1)

Data are *n* (%) unless otherwise specified.

Abbreviations: ECOG PS, Eastern Cooperative Oncology Group performance status; N0, node negative; N1, node positive; PD-L1, programmed cell death ligand 1; TPS, tumour proportion score.

**Figure 1.**
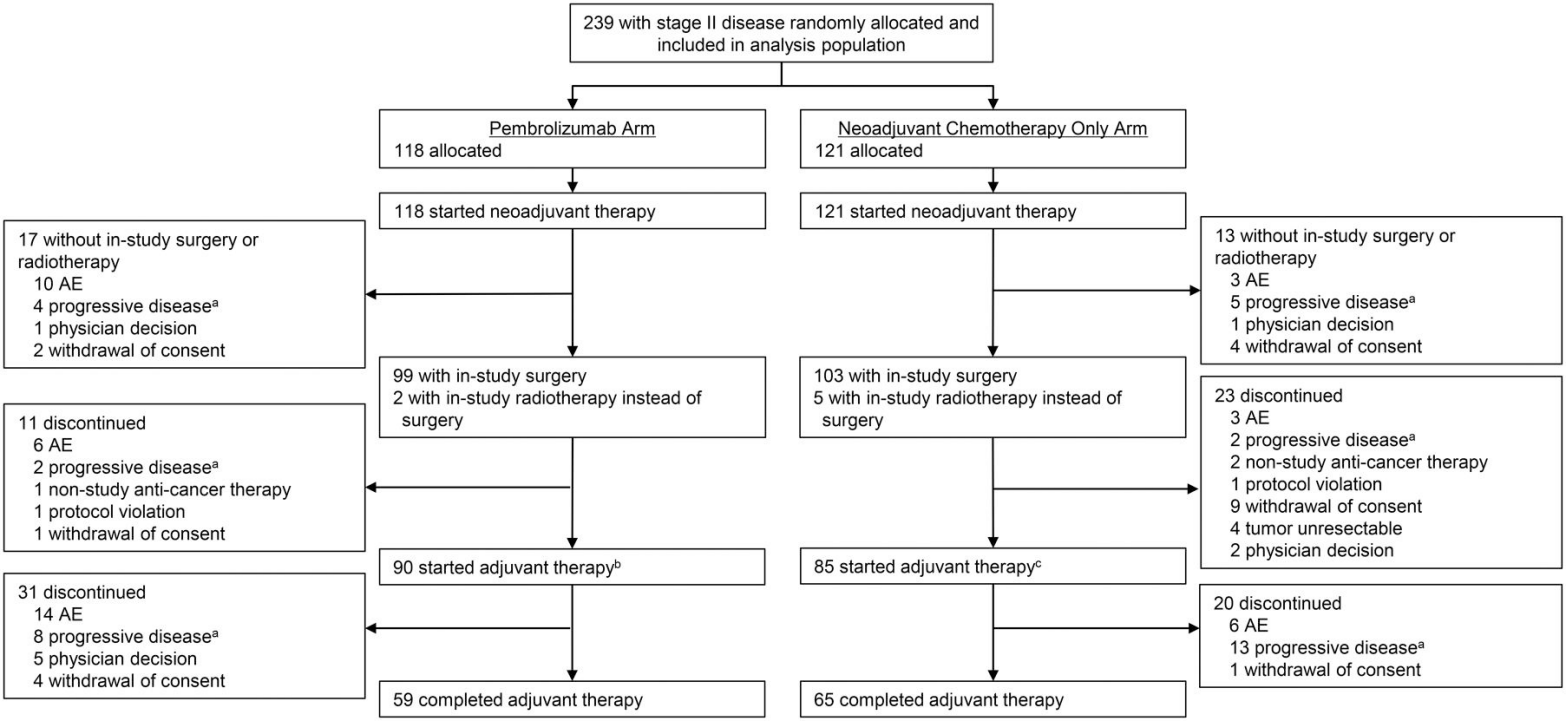
Treatment Disposition. Percentages are based on the number of participants who started neoadjuvant therapy. ^a^Includes clinical progression, local progression preventing surgery, and radiographic progression. ^b^Includes 1 participant who received in-study radiotherapy instead of surgery. ^c^Includes 3 participants who received in-study radiotherapy instead of surgery. Abbreviation: AE, adverse event.

Median (range) time from randomization to data cutoff (August 19, 2024) was 49.9 (32.2-75.3) months. In the pembrolizumab and neoadjuvant chemotherapy only arms, 78 (66.1%) and 91 participants (75.2%), respectively, received 4 cycles of neoadjuvant therapy. A total of 99 (83.9%) and 103 participants (85.1%), respectively, underwent surgery; 90 (76.3%) and 85 (70.2%) started adjuvant therapy (**Figure 1**).

### Surgical outcomes

Among participants who underwent surgery, median (range) time from randomization to surgery was 15.3 (6.7-26.3) weeks in the pembrolizumab arm and 15.6 (4.6-20.6) weeks in the neoadjuvant chemotherapy only arm. Six (6.1%) and 5 participants (4.9%), respectively, had surgical delay, with median (range) time from randomization to surgery of 21.4 (14.9-26.3) weeks in the pembrolizumab arm and 17.6 (13.1-20.6) weeks in the neoadjuvant chemotherapy only arm.

Among those who underwent surgery, lobectomy was performed in 73/99 participants (73.7%) in the pembrolizumab arm and 75/103 (72.8%) in the neoadjuvant chemotherapy only arm (**Table 2**). In the pembrolizumab arm, 94 participants (94.9%) had an R0 resection, and in the neoadjuvant chemotherapy only arm, 89 (86.4%). The most common reasons for not undergoing surgery or radiotherapy (17 in the pembrolizumab arm and 13 in the neoadjuvant chemotherapy only arm) were AEs (pembrolizumab: *n* = 10, 8.5%; neoadjuvant chemotherapy only: *n* = 3, 2.5%) and progressive disease (*n* = 4, 3.4%; *n* = 5, 4.1%, respectively; **Figure 1**).

**Table 2.** Surgical Outcomes

	**Pembrolizumab arm** **(*n* = 99)**	**Neoadjuvant chemotherapy only arm** **(*n* = 103)**
Surgical procedure		
Bilobectomy	11 (11.1)	13 (12.6)
Lobectomy	73 (73.7)	75 (72.8)
Pneumonectomy	15 (15.2)	12 (11.7)
Thoracotomy and biopsy	0 (0.0)	3 (2.9)
Surgical completeness		
R0 resection	94 (94.9)	89 (86.4)
R1 resection	4 (4.0)	10 (9.7)
R2 resection	1 (1.0)	0 (0.0)
Unresectable	0 (0.0)	4 (3.9)
Participants with surgical delay^a^	6 (6.1)	5 (4.9)

Data are *n* (%).

a Defined as >20 weeks between the first neoadjuvant dose and surgery (4 neoadjuvant therapy cycles) or >8 weeks between the last neoadjuvant dose and surgery (1-3 neoadjuvant cycles).

### Efficacy

**Figure 2.**
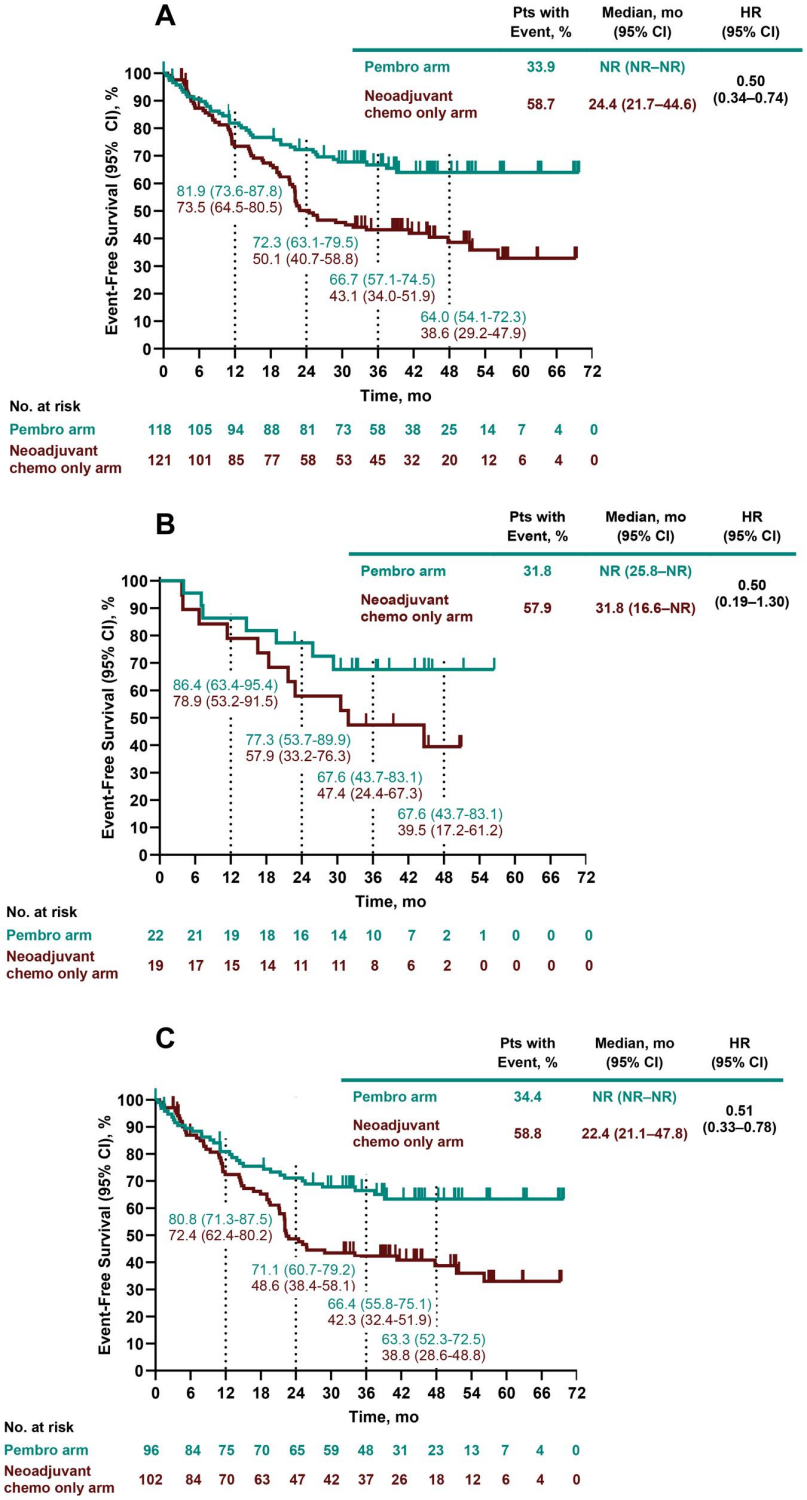
Event-free survival in participants with clinical stage (A) II, (B) IIA, and (C) IIB NSCLC. Abbreviations: Chemo, chemotherapy; EFS, event-free survival; HR, hazard ratio; NR, not reached; NSCLC, non-small-cell lung cancer; Pembro, pembrolizumab.

In the pembrolizumab arm, 40/118 participants (33.9%) experienced an EFS event versus 71/121 participants (58.7%) in the neoadjuvant chemotherapy only arm (HR, 0.50; 95% CI, 0.34-0.74; **Figure 2A**). Median (95% CI) EFS was not reached (NR) in the pembrolizumab arm and was 24.4 (21.7-44.6) months in the neoadjuvant chemotherapy only arm. At 48 months, EFS rates (95% CIs) were 64.0% (54.1%-72.3%) and 38.6% (29.2%-47.9%), respectively. Median EFS, HRs, and 48-month EFS rates in participants with stage IIA and stage IIB disease were consistent with the overall stage II population (**Figure 2B and C**). Event-free survival HR (95% CI) was 0.56 (0.35-0.88) for participants with node-negative (N0) stage II disease and 0.39 (0.19-0.82) for node-positive (N1) stage II disease. Hazard ratios for EFS in additional baseline subgroups are shown in **Figure S1**.

**Figure 3.**
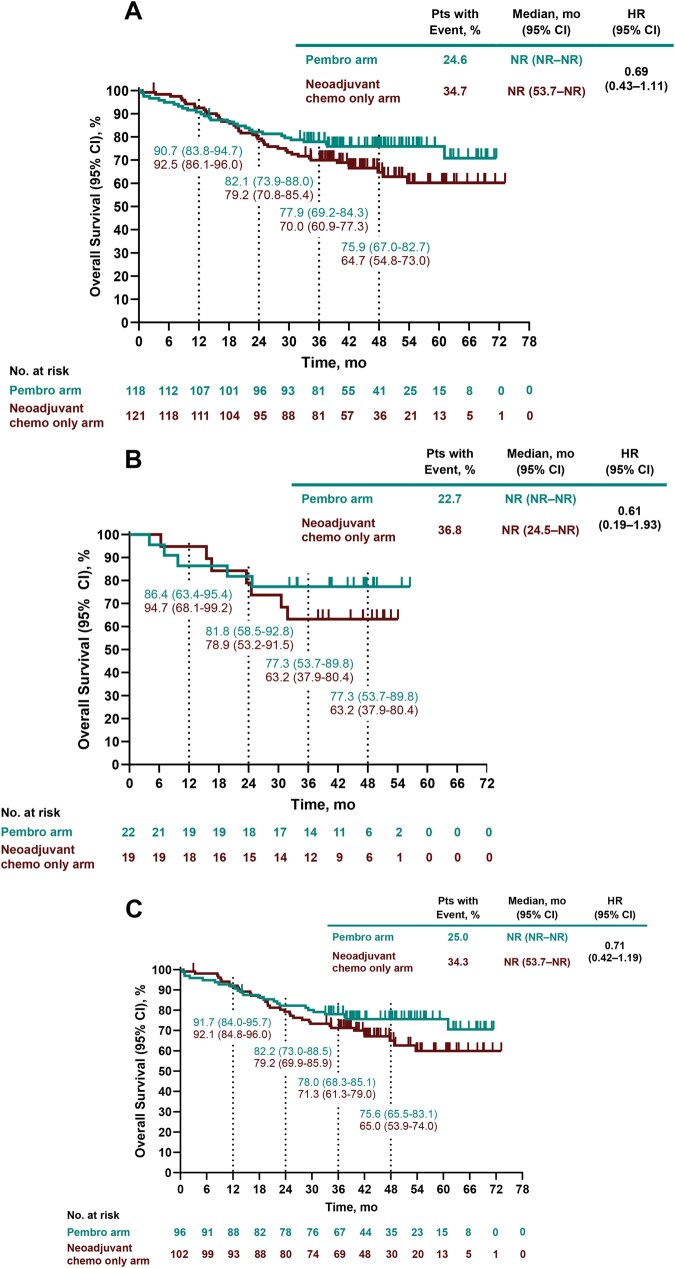
Overall survival in participants with clinical stage (A) II, (B) IIA, and (C) IIB NSCLC. Abbreviations: Chemo, chemotherapy; HR, hazard ratio; NR, not reached; NSCLC, non-small-cell lung cancer; OS, overall survival; Pembro, pembrolizumab.

A total of 29/118 participants (24.6%) in the pembrolizumab arm and 42/121 (34.7%) in the neoadjuvant chemotherapy only arm died by data cutoff (HR, 0.69; 95% CI, 0.43-1.11; **Figure 3A**). Median OS was NR in both arms. The 48-month OS rates (95% CIs) were 75.9% (67.0%-82.7%) and 64.7% (54.8%-73.0%) in the respective arms. Median OS, HRs, and 48-month OS rates in participants with stage IIA and stage IIB disease were consistent with the overall stage II population (**Figure 3B and C**). Overall survival HR (95% CI) was 0.73 (0.42-1.25) for participants with node-negative stage II disease and 0.58 (0.21-1.56) for participants with node-positive stage II disease. Overall survival HRs in additional baseline subgroups are shown in **Figure S2**, and Kaplan-Meier estimates of OS in participants who did not have in-study surgery are shown in **Figure S3**.

**Figure 4.**
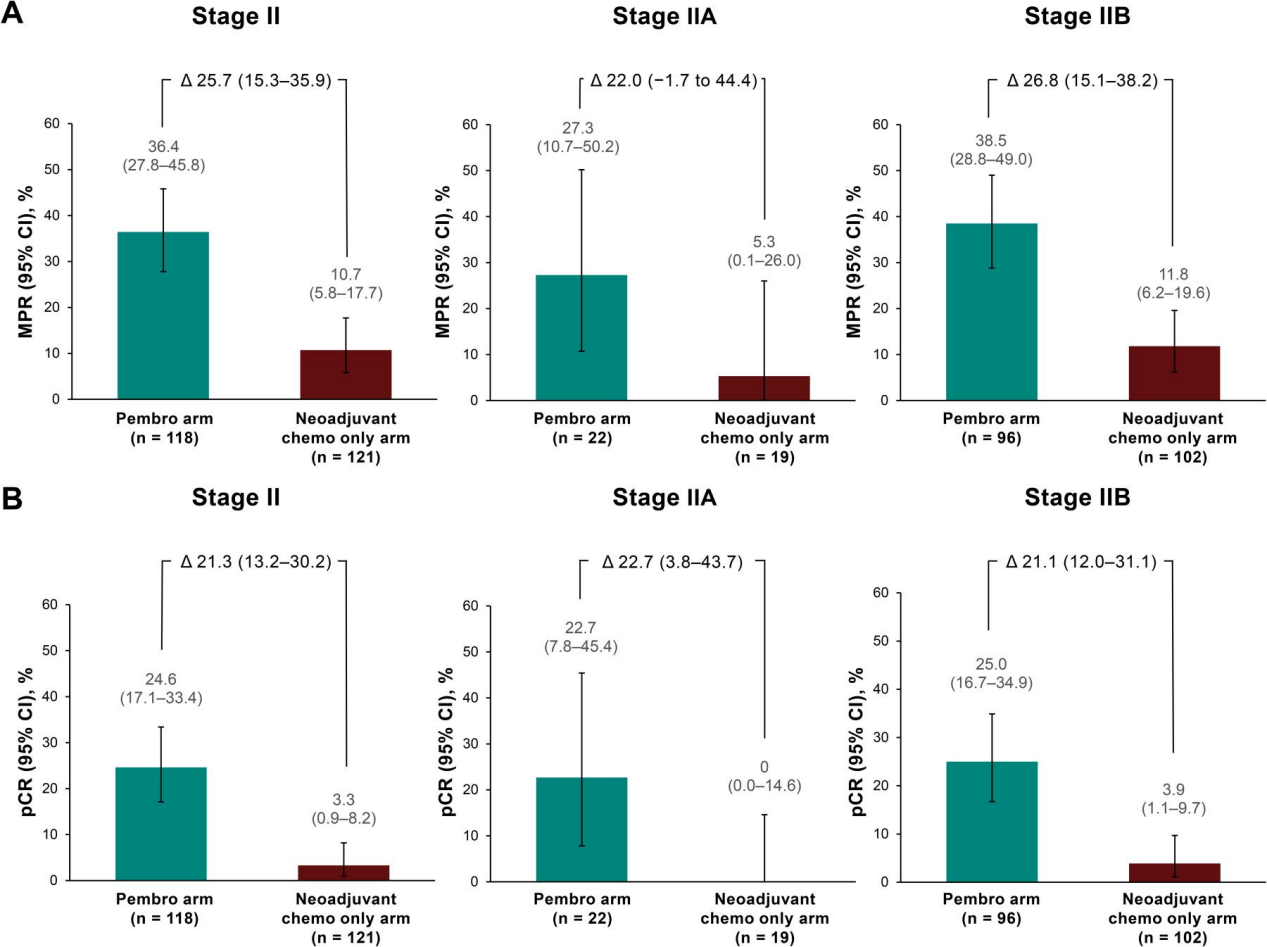
(A) MPR and (B) pCR in participants with clinical stage II, IIA, and IIB NSCLC. Abbreviations: Chemo, chemotherapy; MPR, major pathologic response; NSCLC, non-small-cell lung cancer; pCR, pathologic complete response; Pembro, pembrolizumab.

At the time of surgery, 43/118 participants (36.4%; 95% CI, 27.8%-45.8%) in the pembrolizumab arm and 13/121 participants (10.7%; 95% CI, 5.8%-17.7%) in the neoadjuvant chemotherapy only arm had MPR (percentage difference, 25.7; 95% CI, 15.3-35.9; **Figure 4A**). In the pembrolizumab arm, 29/118 participants (24.6%; 95% CI, 17.1%-33.4%) achieved pCR at the time of surgery versus 4/121 participants (3.3%; 95% CI, 0.9%-8.2%) in the neoadjuvant chemotherapy only arm (percentage difference, 21.3; 95% CI, 13.2-30.2; **Figure 4B**). Both MPR and pCR rates in participants with stage IIA and stage IIB disease were consistent with the overall stage II population (**Figure 4A and B**).

### Safety

Overall, participants received a median of 15 (range, 1-17) doses of pembrolizumab or placebo during the combined neoadjuvant and adjuvant phases.

In the combined neoadjuvant and adjuvant phases, 116/118 participants (98.3%) treated with pembrolizumab plus chemotherapy and 116/121 participants (95.9%) treated with chemotherapy only experienced a treatment-related AE (**Table 3**), which were grade 3 or 4 in 59 participants (50.0%) in the pembrolizumab arm and 49 (40.5%) in the neoadjuvant chemotherapy only arm. Serious treatment-related AEs occurred in 23 (19.5%) and 19 participants (15.7%), respectively. Treatment-related AEs that led to discontinuation of all study treatment occurred in 20 (16.9%) and 9 participants (7.4%) in the respective arms. There were no deaths due to treatment-related AEs.

**Table 3.** Adverse Event Summary

AE	**Pembrolizumab arm** **(*n* = 118)**	**Neoadjuvant chemotherapy only arm** **(*n* = 121)**
Treatment-related AEs, any grade	116 (98.3)	116 (95.9)
Grade 3 or 4	59 (50.0)	49 (40.5)
Serious	23 (19.5)	19 (15.7)
Led to discontinuation of all study treatment	20 (16.9)	9 (7.4)
Led to death	0 (0.0)	0 (0.0)
Immune-mediated AEs and infusion reactions^a^	34 (28.8)	13 (10.7)
Grade 3 or 4	10 (8.5)	5 (4.1)
Serious	7 (5.9)	5 (4.1)
Led to discontinuation of all study treatment	8 (6.8)	3 (2.5)
Led to death	0 (0.0)	0 (0.0)

Data are *n* (%).

a Based on a list of preferred terms intended to capture known risks of pembrolizumab and considered regardless of attribution to study treatment by the investigator.

Abbreviation: AE, adverse event.

Immune-mediated AEs and infusion reactions, regardless of attribution to study treatment, occurred in 34 participants (28.8%) in the pembrolizumab arm and 13 (10.7%) in the neoadjuvant chemotherapy only arm (**Table 3**). Grade 3 or 4 immune-mediated AEs and infusion reactions occurred in 10 (8.5%) and 5 participants (4.1%), and serious immune-mediated AEs and infusion reactions occurred in 7 (5.9%) and 5 (4.1%), respectively. Events leading to discontinuation of all study treatment occurred in 8 (6.8%) and 3 participants (2.5%), respectively. No immune-mediated AEs or infusion reactions led to death. A summary of safety during the neoadjuvant phase is included in **Table S1**.

## Discussion

Consistent with the overall study results of KEYNOTE-671, neoadjuvant pembrolizumab plus chemotherapy followed by surgery and adjuvant pembrolizumab improved clinical outcomes versus neoadjuvant chemotherapy and surgery alone in stage II NSCLC after 4 years of follow-up. Improvements in EFS, OS, MPR, and pCR were observed with perioperative pembrolizumab plus neoadjuvant chemotherapy versus neoadjuvant chemotherapy and surgery alone, along with manageable safety in participants with stage II disease.

A similar proportion of participants with stage II disease underwent surgery in the pembrolizumab (83.9%) and neoadjuvant chemotherapy only arms (85.1%), indicating that combining pembrolizumab with neoadjuvant chemotherapy did not markedly impact participants undergoing surgery. The most common reason for not undergoing surgery or radiotherapy was AEs in the pembrolizumab arm (8.5%) and progressive disease in the neoadjuvant chemotherapy only arm (4.1%). The surgical rates in this study are similar to other phase 3 studies of perioperative/neoadjuvant anti-PD-(L)1 therapies in resectable NSCLC, including in participants with stage II NSCLC (AJCC, eighth edition) in the AEGEAN study (84.3% with neoadjuvant durvalumab plus chemotherapy vs 88.9% with neoadjuvant chemotherapy alone),^10^ and participants with stage IB to II (AJCC, seventh edition) in CheckMate 816 (84.6% with neoadjuvant nivolumab plus chemotherapy vs 83.9% with neoadjuvant chemotherapy alone).^6^ In this analysis, the addition of pembrolizumab to neoadjuvant chemotherapy did not affect choice of surgical procedure or delay surgery. Furthermore, a higher percentage of participants treated with pembrolizumab plus chemotherapy had R0 resections versus chemotherapy only (94.9% vs 86.4%). This is consistent with the overall study population (R0 resection rate of 92.0% vs 84.2% in the pembrolizumab vs neoadjuvant chemotherapy only arm).^11^ These data provide evidence that the addition of pembrolizumab does not adversely impact surgical outcomes in patients with stage II NSCLC.

Perioperative pembrolizumab was associated with clinical benefits in all efficacy outcomes analysed in participants with stage II disease, including EFS, OS, MPR, and pCR, similar to the overall study population^1^^,^^8^ and consistent regardless of whether participants had stage IIA or stage IIB disease. Event-free survival and OS benefits were also observed with pembrolizumab regardless of nodal status at baseline and in most other baseline subgroups evaluated.

Analyses of participants with stage II disease in other phase 3 studies evaluating perioperative anti-PD-(L)1 therapies in resectable NSCLC have been mixed.^2^^,^^3^^,^^5^ In AEGEAN, perioperative durvalumab was associated with improved EFS (HR, 0.76; 95% CI, 0.43-1.34), MPR (percentage difference, 23.7; 95% CI, 12.8-34.6), and pCR (percentage difference, 16.6; 95% CI, 8.1-26.0) versus neoadjuvant chemotherapy alone in stage II disease.^2^ Similarly, in CheckMate 77T, improvements were seen with perioperative nivolumab versus neoadjuvant chemotherapy alone in participants with stage II disease (EFS: HR, 0.81; 95% CI, 0.46-1.43; MPR: percentage difference, 25.9; 95% CI, 12.7-38.1; pCR: percentage difference, 25.9; 95% CI, 14.9-36.9). For both of these studies, EFS outcomes in stage II disease were not as favourable as stage III disease.^2^^,^^3^ EFS results from CheckMate 77T were consistent with those in CheckMate 816, in which neoadjuvant nivolumab had an HR of 0.87 (95% CI, 0.48-1.56) versus chemotherapy alone in stage IB or II NSCLC (AJCC seventh edition) versus 0.54 (95% CI, 0.37-0.80) in stage IIIA NSCLC.^6^ Perioperative tislelizumab was associated with benefits versus neoadjuvant chemotherapy alone in participants with stage II NSCLC in RATIONALE-315 (EFS: HR, 0.47; 95% CI, 0.26-0.87; MPR: percentage difference, 34.4; 95% CI, 21.6-47.2; pCR: percentage difference, 33.3; 95% CI, 22.4-44.2).^5^ Moreover, a meta-analysis of studies evaluating (neo)adjuvant immunotherapy plus chemotherapy in resectable NSCLC showed significantly improved EFS versus neoadjuvant chemotherapy alone.^12^ Together, these findings support the use of perioperative immunotherapy plus neoadjuvant chemotherapy in patients with stage II disease and suggest that perioperative pembrolizumab may provide particularly favourable EFS outcomes.

Results of OS from studies of perioperative immunotherapies are more limited. RATIONALE-315 recently presented significant OS outcomes in the tislelizumab arm (HR, 0.65; 95% CI, 0.45-0.93).^13^ Interim OS results in the overall study populations of AEGEAN and CheckMate 77T showed numerical trends favouring the durvalumab arm (HR, 0.89; 95% CI, 0.70-1.14)^14^ and nivolumab arm (HR, 0.85; 97.63% CI, 0.58-1.25)^15^ versus the neoadjuvant chemotherapy only arm; OS results in participants with stage II disease from these studies are not yet available. Ongoing analyses of OS results will provide further insight into long-term outcomes in patients with stage II disease.

In the current analysis, AEs were manageable after 4 years of follow-up. There were 116 participants (98.3%) with treatment-related AEs with pembrolizumab plus chemotherapy and 116 (95.9%) with chemotherapy only, similar to the overall study population.^1^ There were no deaths due to treatment-related AEs or immune-mediated AEs or infusion reactions among participants with stage II disease. Other phase 3 studies have also shown manageable safety with perioperative immunotherapy plus neoadjuvant chemotherapy in resectable NSCLC.^2–4^

Limitations of the current analysis include that it was exploratory, and subgroup analyses were not adequately powered to detect differences between the treatment arms. Furthermore, the number of participants with stage IIA disease was small, and results from this subgroup should be interpreted with caution.

## Conclusion

The efficacy and safety of perioperative pembrolizumab plus neoadjuvant chemotherapy in participants with stage II NSCLC showed consistent treatment benefits with the overall KEYNOTE-671 population. In addition, surgical outcomes in participants with stage II disease were consistent with the overall population, with the addition of neoadjuvant pembrolizumab to chemotherapy showing no obvious negative impact on surgical outcomes. These findings support the use of perioperative pembrolizumab in patients with stage II NSCLC.

## Supplementary Material

ezag028_Supplementary_Data

## Data Availability

The data sharing policy, including procedures and restrictions, of Merck Sharp & Dohme LLC, a subsidiary of Merck & Co., Inc., Rahway, NJ, USA (MSD) is available at https://externaldatasharing-msd.com/.
