# Supplementary material for: A Subgroup Analysis of Perioperative Pembrolizumab in Clinical Stage II Non-Small-Cell Lung Cancer from the Randomized KEYNOTE-671 Study
Source: Eur J Cardiothorac Surg. 2026 Mar 24;68(3):ezag028. doi: 10.1093/ejcts/ezag028 (PMC13020334; doi:10.1093/ejcts/ezag028)
Supplement: ezag028_Supplementary_Data [file ezag028_supplementary_data.docx]

# SUPPLEMENTARY MATERIAL

## Supplementary Methods

### Surgical treatment

Surgery was performed ≤20 weeks after the first neoadjuvant dose; participants who received ≤4 cycles of neoadjuvant therapy underwent surgery within 4 to 8 weeks of the last neoadjuvant dose. Surgical delay was defined as surgery performed >20 weeks after the first neoadjuvant dose (4 neoadjuvant therapy cycles) or >8 weeks after the last neoadjuvant dose (1‒3 neoadjuvant therapy cycles).

Surgery was lobectomy, bilobectomy, pneumonectomy, sleeve lobectomy, sleeve pneumonectomy, or chest wall resection with any of the previously listed resections. Wedge resection or segmentectomy was not permitted. Lymph node levels were defined according to the criteria of the joint AJCC/UICC classification in the IASLC Staging Manual (2^nd^ edition), and a detailed description of lymph node dissection including nodal levels and number removed were documented in the operative note. Preferred mediastinal lymph node (N2) dissection consisted of complete removal of all accessible ipsilateral mediastinal lymph node levels. Acceptable lymph node dissection included removal of at least 2 N2 levels, one of which was Level 7. If a lymph node level was explored, and no lymph nodes were present, these findings were documented. Additionally, all lymph nodes with documented metastatic disease prior to neoadjuvant therapy were removed.

Participants who did not undergo in-study surgery for any reason except local progression or metastatic disease could receive radiotherapy and continue to the adjuvant phase of the study. Participants with microscopic or gross residual disease after surgery, including extracapsular nodal extension, also received radiotherapy. Treatment continued until the maximum number of doses was reached, disease progression or recurrence, unacceptable toxicity, or participant withdrawal.

Supplementary Table S1. Adverse Event Summary in the Neoadjuvant Treatment Phase^a^

| **AE** | Pembrolizumab Arm  **(n=118)** | **Neoadjuvant Chemotherapy Only Arm**  **(n=121)** |
| --- | --- | --- |
| Treatment-related AEs, any grade | 114 (96.6) | 115 (95.0) |
| Grade 3 or 4 | 53 (44.9) | 45 (37.2) |
| Serious | 17 (14.4) | 14 (11.6) |
| Led to discontinuation of all study treatment | 6 (5.1) | 4 (3.3) |
| Led to death | 0 (0.0) | 0 (0.0) |
| Immune-mediated AEs and infusion reactions^b^ | 12 (10.2) | 5 (4.1) |
| Grade 3 or 4 | 5 (4.2) | 1 (0.8) |
| Serious | 3 (2.5) | 0 (0.0) |
| Led to discontinuation of all study treatment | 3 (2.5) | 0 (0.0) |
| Led to death | 0 (0.0) | 0 (0.0) |

Data are n (%).

AE, adverse event.

^a^Includes AEs in the neoadjuvant phase and, if the participant did not receive adjuvant pembrolizumab or placebo, AEs up to 30 days after the last dose of neoadjuvant treatment (90 days for serious AEs).

^b^Based on a list of preferred terms intended to capture known risks of pembrolizumab and considered regardless of attribution to study treatment by the investigator.

Supplementary Figure S1. EFS by subgroup in participants with clinical stage II NSCLC.


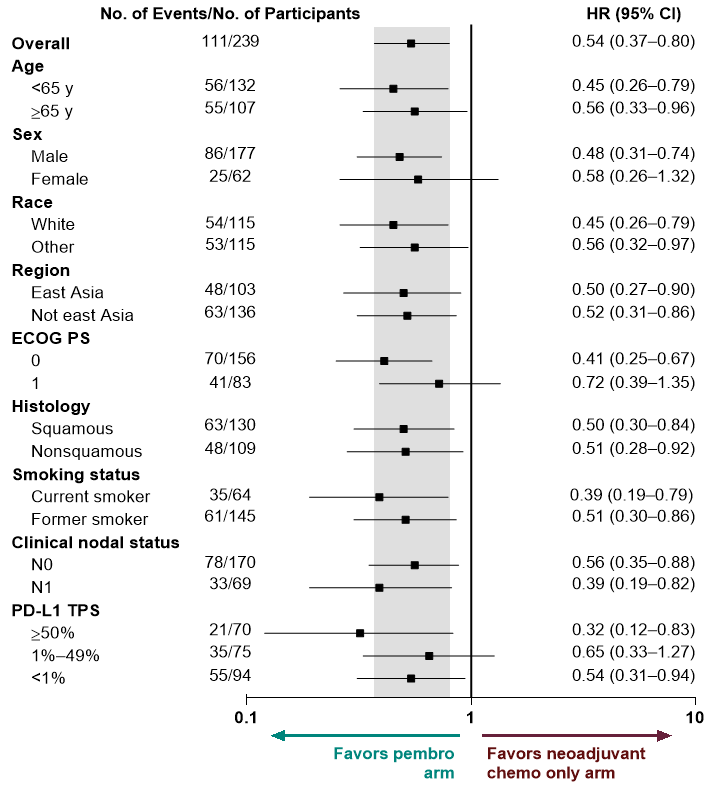


Chemo, chemotherapy; ECOG PS, Eastern Cooperative Oncology Group performance status; HR, hazard ratio; N0, node negative; N1, node positive; NSCLC, non‒small-cell lung cancer; PD-L1, programmed cell death ligand 1; Pembro, pembrolizumab; TPS, tumor proportion score.

Supplementary Figure S2. OS by subgroup in participants with clinical stage II NSCLC.


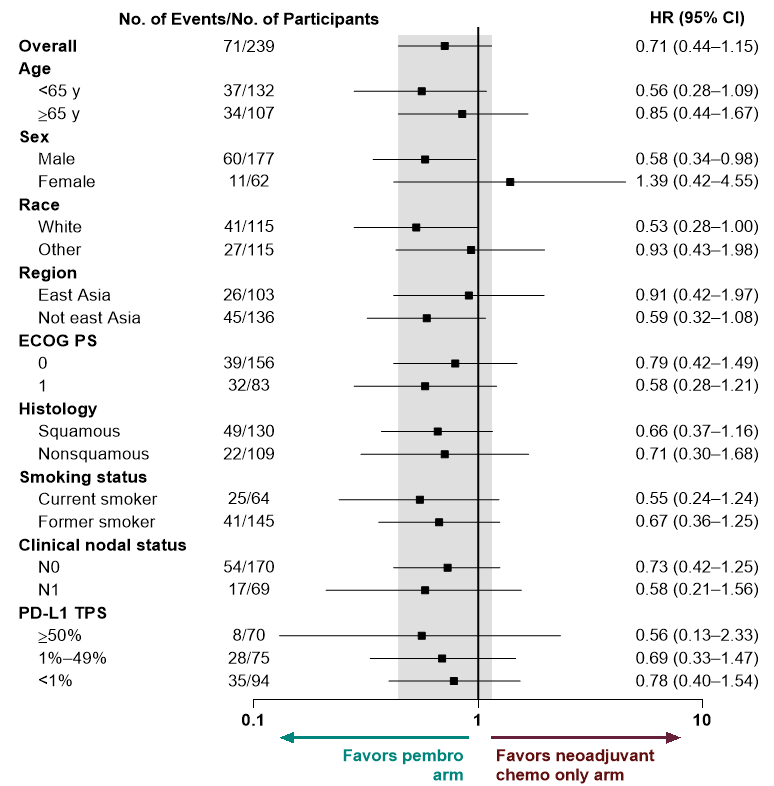


Chemo, chemotherapy; ECOG PS, Eastern Cooperative Oncology Group performance status; HR, hazard ratio; N0, node negative; N1, node positive; NSCLC, non‒small-cell lung cancer; PD-L1, programmed cell death ligand 1; Pembro, pembrolizumab; TPS, tumor proportion score.

Supplementary Figure S3. OS in participants who did not have surgery.


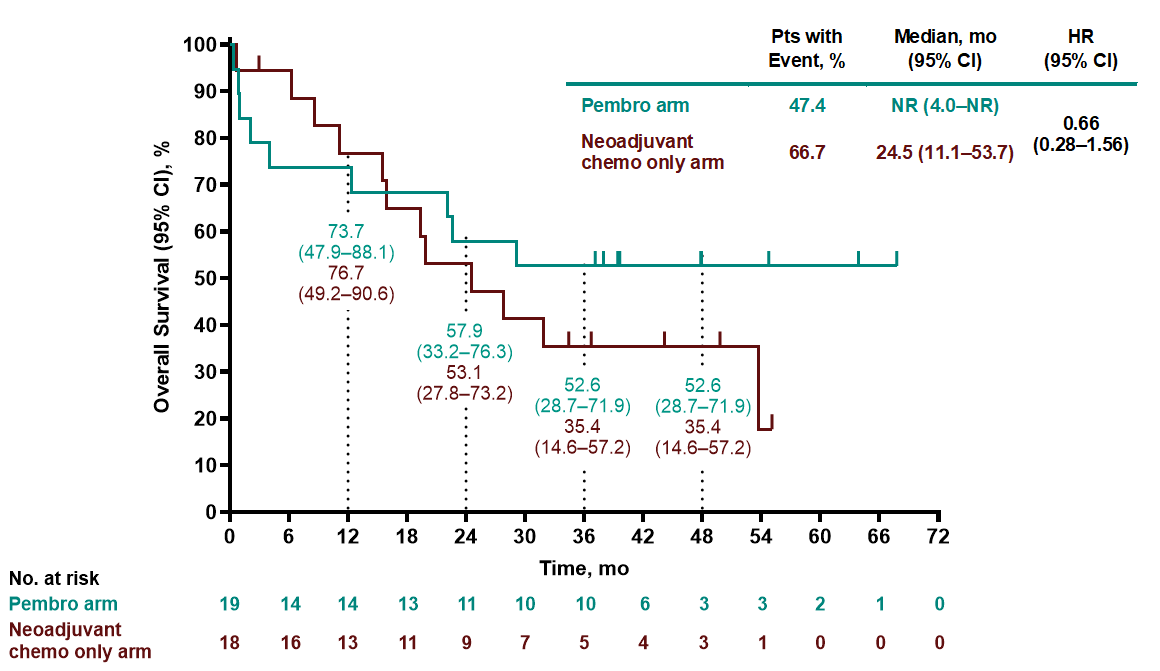


Chemo, chemotherapy; HR, hazard ratio; NR, not reached; OS, overall survival; Pembro, pembrolizumab.

# ACKNOWLEDGMENTS

We thank the participants and their families and caregivers for participating in this study, along with all investigators and site personnel. Medical writing assistance was provided by Tina Nie, PhD, of ICON plc (Blue Bell, PA, USA), funded by Merck Sharp & Dohme LLC, a subsidiary of Merck & Co., Inc., Rahway, NJ, USA.

# DATA AVAILABILITY STATEMENT

Merck Sharp & Dohme LLC, a subsidiary of Merck & Co., Inc., Rahway, NJ, USA (MSD) is committed to providing qualified scientific researchers access to anonymized data and clinical study reports from the company’s clinical trials for the purpose of conducting legitimate scientific research. MSD is also obligated to protect the rights and privacy of trial participants and, as such, has a procedure in place for evaluating and fulfilling requests for sharing company clinical trial data with qualified external scientific researchers. The MSD data sharing website (available at: <https://externaldatasharing-msd.com/>) outlines the process and requirements for submitting a data request. Applications will be promptly assessed for completeness and policy compliance. Feasible requests will be reviewed by a committee of MSD subject matter experts to assess the scientific validity of the request and the qualifications of the requestors. In line with data privacy legislation, submitters of approved requests must enter into a standard data-sharing agreement with MSD before data access is granted. Data will be made available for request after product approval in the US and EU or after product development is discontinued. There are circumstances that may prevent MSD from sharing requested data, including country or region-specific regulations. If the request is declined, it will be communicated to the investigator. Access to genetic or exploratory biomarker data requires a detailed, hypothesis-driven statistical analysis plan that is collaboratively developed by the requestor and MSD subject matter experts; after approval of the statistical analysis plan and execution of a data-sharing agreement, MSD will either perform the proposed analyses and share the results with the requestor or will construct biomarker covariates and add them to a file with clinical data that is uploaded to an analysis portal so that the requestor can perform the proposed analyses.

## Full Conflicts of Interest for Authors

**Masahiro Tsuboi:** Funding to the institution to support study conduct from Merck Sharp & Dohme LLC, a subsidiary of Merck & Co., Inc., Rahway, NJ, USA; funding to the institution from AstraZeneca, AstraZeneca KK, Ono Pharmaceutical Co., LTD, Bristol Myers Squibb KK, Eli Lilly, Novartis, and MiRXES. Advisor for AstraZeneca KK, MSD, Chugai Pharmaceutical Co., LTD., Novartis, Ono Pharmaceutical Co., LTD., Bristol Myers Squibb KK, and MiRXES. Consultant for Chugai Pharmaceutical Co., LTD. Speaker for Johnson & Johnson Japan (Ethicon), AstraZeneca KK, Eli Lilly Japan, Chugai Pharmaceutical Co., LTD, Taiho Pharma, Medtronic Japan, Ono Pharmaceutical Co., LTD, MSD, Bristol Myers Squibb KK, Daiichi-Sankyo, and Amgen KK

**Heather Wakelee:** Research funding to the institution from Bayer, AstraZeneca, BMS, Genentech/Roche, MSD, Helsinn, Pfizer (formerly SeaGen), Gilead (via IIT) and Xcovery; serving as a compensated advisory board member for IOBiotech, OncoC4, GSK and BeOne (formerly BeiGene); serving as an uncompensated advisory board member for MSD, Genentech/Roche, BMS, and AstraZeneca; serving on the executive committee of ECOG-ACRIN.

**Marina C. Garassino:** Consulting fees from AstraZeneca, Abion, MSD International GmbH, Bayer, BMS, Boehringer Ingelheim Italia S.p.A, Celgene, Eli Lilly, Incyte, Novartis, Pfizer, Roche, Takeda, Seattle Genetics, Mirati, Daiichi Sankyo, Regeneron, Merck & Co., Inc., Rahway, NJ, USA, Blueprint, Janssen, Sanofi, AbbVie, BeiGene, Oncohost, Medscape, Gilead, lo Biotech, and Revolution Medicines; payment or honoraria for lectures from AstraZeneca, Merck & Co., Inc., Rahway, NJ, USA, Daiichi Sankyo, Gilead, Eli Lilly, and Regeneron; and support for attending meetings or travel from AstraZeneca.

**Shugeng Gao:** Funding to the institution to support study conduct from Merck Sharp & Dohme LLC, a subsidiary of Merck & Co., Inc., Rahway, NJ, USA.

**Alexander Luft:** None declared.

**Ke-Neng Chen**: Funding to the institution to support study conduct from Merck Sharp & Dohme LLC, a subsidiary of Merck & Co., Inc., Rahway, NJ, USA.

**Jonathan D Spicer:** Grants to the institution from AstraZeneca, MSD, Roche, BMS, CLS Therapeutics, Protalix Biotherapeutics, Pfizer, and Regeneron; consulting fees from AstraZeneca, MSD, Roche, BMS, Novartis, Chemocentryx, Amgen, Protalix Biotherapeutics, Xenetic Biosciences, Regeneron, Eisai, and Pfizer; payment for a speaking role from Peerview, OncLive, and Medscape; support for attending meetings or travel from AstraZeneca, Merck, and BMS; participating on a clinical trial safety monitoring board for AstraZeneca; and receiving equipment, materials, drugs, gifts, or other services via grant to the institution from Roche, MSD, BMS, and AstraZeneca.

**Yuming Zhu:** Funding to the institution to support study conduct from Merck Sharp & Dohme LLC, a subsidiary of Merck & Co., Inc., Rahway, NJ, USA.

**Hisashi Saji:** Grants to the institution from Chugai-pharm, TAIHO, Boehringer Ingelheim, Lilly, ETHICON, and Covidien; payment for a speaking role from MSD, Boehringer Ingelheim, ETHICON, Covidien, Chugai-pharm, Astellas Pharma, FUJIFILM Medical, Bristol-Myers Squibb, Takeda, AstraZeneca, CSL Behring, and TAIHO.

**Morihito Okada:** None declared.

**Tõnu Vanakesa:** None declared.

**Haiquan Chen:** None declared.

**Guofang Zhao:** None declared.

**Norihiko Ikeda:** Research funding to the department from AstraZeneca, Chugai Pharma, Boehringer Ingelheim, Taiho Pharma, Eli Lilly, Ono Pharma, BMS, MSD, Nihon Mediphysics, Teijin Pharma, Eisai, Daiichi-Sankyo, Fuji film, and Johnson & Johnson; honoraria from AstraZeneca, Chugai Pharma, Boehringer Ingelheim, Taiho Pharma, Eli Lilly, Ono Pharma, BMS, Olympus, MSD, Johnson & Johnson, Nihon Mediphysics, Medtronics, and Teijin Pharma; and served as a past president of the Japan Surgical Society and the Japan Lung Cancer Society.

**David R. Jones:** Funding to the institution to support study conduct from Merck Sharp & Dohme LLC, a subsidiary of Merck & Co., Inc., Rahway, NJ, USA. Consultant for AstraZeneca and More Health. Speaker for Genentech and Peerview.

**Benny Weksler:** Funding to the institution to support study conduct from Merck Sharp & Dohme LLC, a subsidiary of Merck & Co., Inc., Rahway, NJ, USA. Grant or research support from Articure. Consultant for Intuitive Surgery. Speaker for AstraZeneca.

**Chien-Sheng Huang:** Funding to the institution to support study conduct from Merck Sharp & Dohme LLC, a subsidiary of Merck & Co., Inc., Rahway, NJ, USA.

**Moishe Liberman:** Funding to the institution to support study conduct from Merck Sharp & Dohme LLC, a subsidiary of Merck & Co., Inc., Rahway, NJ, USA. Grant or research support from BMS, AstraZeneca, J&J, Ethicon, Olympus, Cook, Intuitive, Pfizer, Roche, Novartis, POINT, Galvanize, and Caprion.
